# Complete chloroplast genomes of three *Pleurozia* species and comparative analyses with *P. purpurea*: codon usage bias and phylogeny

**DOI:** 10.3389/fpls.2025.1599291

**Published:** 2025-07-18

**Authors:** Sheng Bi, Qin Liu, Jie-Wei Hao, Xiang-Zhe Cai, De Gao, Li-Na Zhang

**Affiliations:** ^1^ Ministry of Education Key Laboratory for Genetics and Germplasm Innovation of Tropical Special Forest Trees and Ornamental Plant, School of Life and Health Sciences, Hainan University, Haikou, Hainan, China; ^2^ International Joint Center for Terrestrial Biodiversity around South China Sea of Hainan Province, School of Ecology, Hainan University, Haikou, Hainan, China; ^3^ College of Agricultural Engineering, Guangxi Vocational University of Agriculture, Nanning, Guangxi, China; ^4^ College of Geographical Sciences, Hebei Normal University, Shijiazhuang, Hebei, China

**Keywords:** *Pleurozia*, chloroplast genome, comparative genomics, codon usage bias, phylogeny

## Abstract

The liverwort genus *Pleurozia*, a morphologically specialized bryophyte group, holds unique taxonomic and evolutionary significance. This study sequenced and assembled the chloroplast genomes of three *Pleurozia* species (*P. acinosa*, *P. gigantea*, and *P. subinflata*), with genome sizes of 118,233 bp, 118,423 bp, and 118,304 bp, respectively. All three genomes exhibit the typical quadripartite structure. Comparative genomics analyses, including the genome of *P. purpurea*, revealed high conservation in genome size, gene content, and inverted repeat (IR) boundaries. Coding regions were more conserved than noncoding and intronic regions, suggesting the potential of the latter as molecular markers. The IR regions also displayed significantly lower sequence divergence compared to the single-copy regions. Most protein-coding genes were subject to purifying selection, whereas *ycf66* and *ndhD* showed signs of positive selection. Codon usage bias analyses across the four species identified a consistent preference for U- and A-ending codons, with a moderate bias primarily shaped by natural selection, in conjunction with mutation pressure. Phylogenetic analyses based on 35 liverwort chloroplast genomes strongly supported the monophyly of *Pleurozia* and confirmed Pleuroziales as an evolutionary intermediate between thalloid and leafy liverworts. These findings provide valuable genomic resources for improving our understanding of species delimitation, phylogenetic relationships, and evolutionary mechanisms in liverworts.

## 1 Introduction

Liverworts (Marchantiophyta), comprising approximately 7,300 species (Söderström et al., 2016), represent a key lineage in land plant evolution. Their pivotal evolutionary role is supported by both fossil records (Wellman et al., 2003; Rubinstein et al., 2010; Clarke et al., 2011) and phylogenetic analyses (Bowman, 2013; Cox et al., 2014; Li et al., 2024). Current phylogenetic studies reveal an evolutionary trajectory in liverworts from complex thalloid via simple thalloid to leafy conditions (Forrest et al., 2006; Qiu et al., 2006). Within this evolutionary framework, the genus *Pleurozia* Dumort. (Pleuroziales: Pleuroziaceae) occupies a significant position hypothesized to be a transitional group bridging simple thalloid and leafy liverworts (He-Nygrén et al., 2006). *Pleurozia* is notable for its distinctive two-sided apical cell, differing from the typical three-sided apical cell found in other leafy liverworts (Crandall-Stotler, 1976; Thiers, 1993). Furthermore, this genus also displays diverse morphological features, notably the formation of sac-shaped leaves and the frequent occurrence of sterile perianths. These unique traits not only demonstrate the genus’s evolutionary distinctiveness but also enhance its value for understanding liverwort phylogeny and evolutionary processes. Ecologically, *Pleurozia* species are predominantly epiphytic, primarily inhabiting high-elevation tropical and subtropical montane rainforests (Thiers, 1993)—habitats that are particularly sensitive to environmental disturbances such as climate change. This niche specialization renders *Pleurozia* not only a potential bioindicator for high-altitude forest ecosystems, but also highly vulnerable to environmental threats.

There are twelve species in *Pleurozia* according to the latest taxonomic checklist (Söderström et al., 2016). However, the placement of Pleuroziaceae within the liverwort taxonomic framework, as well as the resolution of its infrageneric relationships, has been historically challenging when based solely on morphological data (He-Nygrén et al., 2006), likely due to the reduction and subsequent re-evolution of morphological characters within the lineage (Thiers, 1993). DNA barcoding, first proposed by Hebert et al. (2003), has become a valuable tool for taxonomic studies, particularly in resolving ambiguous identifications, uncovering cryptic species, identifying new taxa, and reconstructing phylogenetic relationships (Hollingsworth et al., 2009; Krawczyk et al., 2014; Bączkiewicz et al., 2017). However, its effectiveness varies across plant groups and has shown notable limitations in liverworts (Ślipiko et al., 2020). The advent of Next-Generation Sequencing (NGS) has made complete plastid genomes (plastomes) an increasingly powerful alternative. Complete plastid genomes now serve as powerful molecular markers for classification, phylogenetic reconstruction, and evolutionary studies across diverse land plants (Dodsworth, 2015; Li et al., 2015). Their utility extends to distinguishing closely related taxa (e.g., Szczecińska and Sawicki, 2015; Myszczyński et al., 2017; Krawczyk et al., 2018), varieties, and individual genotypes (Kane et al., 2012), significantly improving phylogenetic resolutions across various taxonomic levels (e.g., Zhang et al., 2011; Wang et al., 2016; Chen et al., 2022). Although the first liverwort chloroplast genome (*Marchantia polymorpha* L.) was sequenced over three decades ago (Ohyama et al., 1986), the number of complete liverwort genomes in GenBank remains strikingly low compared to vascular plants. This scarcity substantially hinders our understanding of plastid genome evolution in bryophytes, limits the application of plastid sequences in comprehensive phylogenomic analyses (Wang et al., 2022; Li et al., 2024), and critically impedes efforts to resolve the phylogenetic placement of key transitional taxa such as *Pleurozia*. Moreover, the presumed conserved quadripartite structure of liverwort plastomes (Yu et al., 2019; Dong et al., 2021) requires further validation through expanded taxonomic sampling.

Although *Pleurozia* occupies a unique phylogenetic position, its study has been hindered by challenges in specimen collection and preservation, resulting in limited data availability for modern taxonomic revisions and molecular phylogenetic analyses. To date, only the complete plastid genome of *P. purpurea* and *P. subinflata* have been sequenced and preliminarily analyzed (Wang et al., 2009; Dong et al., 2021; Song et al., 2024). In China, five *Pleurozia* species have been recorded (*P. acinosa*, *P. caledonica*, *P. subinflata*, *P. gigantea*, and *P. purpurea*), all restricted to southern regions, with four of these (excluding *P. purpurea*) occurring on Hainan Island (http://www.sp2000.org.cn/). Among these species, *P. purpurea* and *P. subinflata* are categorized as Near Threatened (NT) on the China Biodiversity Red List: Higher Plants Volume (2020). Additionally, *P. caledonica* has not been reported or re-collected since its initial discovery on Hainan Island in 1998 (Bai and Li, 1998). Therefore, this study aims to enhance the genomic resources for this important genus by sequencing the complete chloroplast genomes of three *Pleurozia* species from Hainan Island. Specifically, our objectives are to: 1) assemble and annotate the complete chloroplast genomes of the three *Pleurozia* species; 2) analyze the chloroplast genome characteristics and codon usage patterns within *Pleurozia*; 3) investigate the phylogenetic placement of *Pleurozia* as a potential transitional lineage between simple thalloid and leafy liverworts.

## 2 Materials and methods

### 2.1 Plant materials and DNA extraction

**Figure 1:**
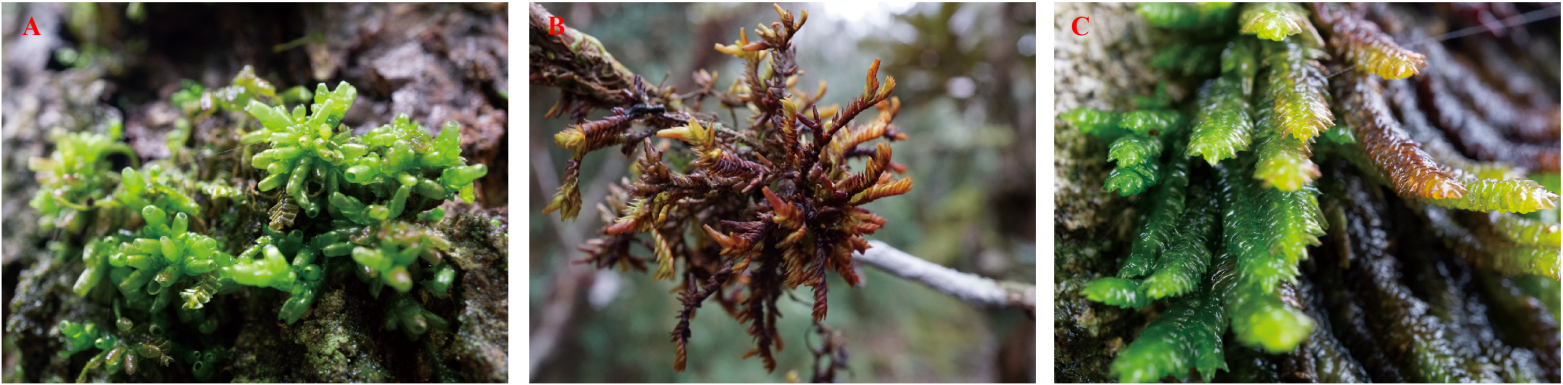
Morphological characteristics and habitats of *Pleurozia* species. **(A)** *P. acinosa*. **(B)** *P. subinflata*. **(C)** *P. gigantea*.

Fresh specimens of three *Pleurozia* species were collected from tree trunks in the tropical cloud forests of Wuzhishan Mountain (108°42′E, 18°53′N), Hainan, China, in October 2022. Their morphological characteristics in the wild is illustrated in **Figure 1**. Voucher specimens have been preserved at the Herbarium of Hainan University (HUTB). Species identification was performed by Li-Na Zhang. Each specimen was meticulously cleaned with distilled water and then dried using absorbent paper. To reduce potential contamination from other plant sources, the clean shoots were isolated using a stereomicroscope and immediately frozen in liquid nitrogen. They were stored in an ultralow-temperature freezer at -80 °C pending further analysis. Total genomic DNA extractions were carried out employing the Universal Genomic DNA Kit (CW2298, CWBIO), and using the Agilent 5400 Fragment Analyzer system, their integrity, quality, and concentration were assessed.

### 2.2 Chloroplast genome sequencing, assembly and annotation

DNA clusters were sequenced on the Illumina NovaSeq 6000 platform, achieving average sequencing depths of 795X for *P. acinosa*, 409X for *P. gigantea*, and 189X for *P. subinflata*. The sequencing produced raw sequences with a read length of 150 bp, including paired-end sequencing of total DNA and the construction of an Illumina PE library. The raw sequencing image data were converted into sequence data via Base Calling and saved in the FASTQ format. These sequences underwent a rigorous quality control process, which included the removal of adapter sequences and 5’-end bases that were not AGCT, trimming reads with quality values below Q20, discarding reads with N proportions of 10% or higher, and eliminating joint sequences and small segments under 75 bp after pruning. Consequently, high-quality read sequences (clean reads) were obtained. For the *de novo* assembly of these clean data, SPAdes v3.14.1 (Prjibelski et al., 2020) was used in “careful” mode with default k-mers, and the sequences were self-corrected using the Hammer algorithm. Following the initial assembly, reassembly was performed with optimized k-mer settings (93, 95, 97, 103, 105, 107, and 115) using VelvetOptimiser (Zerbino and Birney, 2008) to integrate the results.

The extracted chloroplast genome sequences were merged into a single FASTA file after alignment with published chloroplast DNA (cpDNA) data and protein-coding gene (PCG) sequences of closely related species using BLASTn and Exonerate. The PRICE (Paired-Read Iterative Contig Extension) algorithm facilitated iterative contig extension until the sequence length stabilized (Ruby et al., 2013). Subsequently, the original sequencing reads were reviewed, and paired reads were selected for reassembly using Bowtie2 (Langmead and Salzberg, 2012). The circular chloroplast genome was then extracted after performing a final assembly with SPAdes v3.14.1 (Prjibelski et al., 2020).

The initial annotation of chloroplast genomes was performed using PGA (available at https://github.com/quxiaojian/PGA) (Qu et al., 2019), with *Porella perrottetiana* (GenBank accession: NC_043780) and *Ptilidium pulcherrimum* (GenBank accession: HM222519) serving as reference sequences for preliminary annotation. Re-annotation was subsequently performed using Geneious Prime 2023.2.1, utilizing the GenBank file from the initial annotation results and setting a 70% similarity threshold for annotation. Initiation and termination codons, along with intron/exon boundaries, were manually verified by referencing sequences from closely related species. tRNA genes were identified using tRNAscan-SE 2.0.7 (Chan et al., 2021), and rRNA genes were annotated with RNAmmer 1.2 (Lagesen et al., 2007). Finally, the large single-copy (LSC) region, small single-copy (SSC) region, and inverted repeat (IR) regions of the cpDNA were annotated using the Repeats Finder plugin in Geneious Prime 2023.2.1.

The assembled and annotated cp-genome sequences have been deposited in GenBank (http://www.ncbi.nlm.nih.gov/) under the accession numbers OR168937, OR168938 and OR168939.

### 2.3 Characteristic analysis of chloroplast genome

The annotated chloroplast genome maps of the three *Pleurozia* species were generated using the online chloroplast genome mapping tool Chloroplot (Zheng et al., 2020). Further analysis in Geneious Prime 2023.2.1 enabled us to ascertain crucial chloroplast genome characteristics, including the total length, GC content, the number of PCGs, introns, tRNA genes, and rRNA genes.

### 2.4 Comparative genomic analysis and nucleotide diversity

To elucidate the sequence divergence within the chloroplast genomes of *Pleurozia* species, we used a custom Perl script (available at https://github.com/quxiaojian/Bioinformatic_Scripts) to transform the GenBank files into mVISTA-compatible formats. Employing *P. purpurea* cpDNA (GenBank accession: MK645838; also included in subsequent analyses) as a reference, we compared the complete chloroplast genome sequences of the three newly sequenced species using the mVISTA tool in Shuffle-LAGAN mode (Frazer et al., 2004). Structural variations and collinearity within the *Pleurozia* cpDNA were investigated through progressive Mauve alignments in the Mauve software, enabling the creation of a structural variation map (Darling et al., 2004, 2010). A sliding window analysis was conducted to assess nucleotide variability (Pi) across the entire chloroplast genome using DnaSp v6 (Rozas et al., 2017), with a window length of 600 bp and a step size of 200 bp. To analyze the expansion and contraction of the IR regions, the MUMmer4 and CPJSdraw software were employed to delineate the boundaries between the single-copy (SC) and IR regions (Marçais et al., 2018; Li et al., 2023).

### 2.5 Selective pressure analysis

To accurately assess selective pressures in the molecular evolution of chloroplast genomes, homologous protein-coding genes (PCGs) were extracted from the chloroplast genomes of four *Pleurozia* species using Geneious Prime 2023.2.1. The Translation Align module of Geneious Prime 2023.2.1 was employed to select the transl_table 11 genetic code (Bacterial, Archaeal, and Plant Plastid Code) for MAFFT v7.490 alignment (Katoh, 2002; Katoh and Standley, 2013). Subsequently, Ka, Ks, and Ka/Ks values of PCGs were calculated using transl_table 11 with the YN method selected in KaKs_Calculator 3.0 (Yang and Nielsen, 2000; Zhang, 2022; Hu et al., 2023). Here, Ka represents the rate of nonsynonymous substitutions, while Ks indicates the rate of synonymous substitutions. By comparing these substitution rates and the Ka/Ks ratio, inferences were made regarding whether the PCGs are under positive selection (Ka/Ks > 1), negative selection (Ka/Ks < 1), or neutral selection (Ka/Ks = 1). Finally, the R language packages ggplot2 (Wickham, 2016) and aplot (Yu, 2023) were utilized to create bubble charts for visualizing the results.

### 2.6 Analysis of codon usage bias

To explore the codon usage bias (CUB), we analyzed the chloroplast genomes of four *Pleurozia* species. The coding sequences (CDS) were extracted using Geneious Prime 2023.2.1 and screened to ensure correct transcriptional start codons (transl_table 11), with sequences shorter than 300 bp excluded. We calculated GC1, GC2, and GC3 (the G+C content at the first, second and third codon position, excluding stop codons), as well as *P_1_*, *P_2_*, and *P_3_* (G+C content at the first, second and third codon positions, excluding ATG, ATA, TGG and stop codons) (Sueoka, 1999). Additionally, we determined A3s, T3s, C3s, and G3s (the content of A, T, C and G at synonymous third codon positions, excluding ATG, ATT, ATC, ATA, TGG and stop codons) for each chloroplast genomic CDS using Python scripts. The effective number of codons (ENC) (Wright, 1990), relative synonymous codon usage (RSCU) (Sharp and Li, 1986), and GC3s (the G+C content at the third position of synonymous codons) were calculated using the codonW 1.4.2 program. RSCU values reflect the preference or avoidance of synonymous codons, with an RSCU value of 1.0 indicating no bias, while values deviating from 1.0 indicate positive or negative codon preference, respectively (He et al., 2016). ENC values, ranging from 20 to 61, indicate the degree of codon usage bias, with lower values (≤35) suggesting a strong preference (Parvathy et al., 2022).

To investigate the forces shaping CUB, we performed three analyses. First, an ENC-GC3s plot was generated to distinguish the effects of mutation pressure versus natural selection. Genes lying on or near the standard curve are considered to be primarily influenced by mutation pressure, whereas those deviating significantly below the curve are shaped by selection (Wright, 1990). Second, a Parity Rule 2 (PR2) plot was constructed by plotting A3/(A3+T3) against G3/(G3+C3) (Sueoka, 1995). Deviation of genes from the central point (0.5, 0.5), where A=T and G=C, indicates the relative influence of selection over mutation pressure (Chakraborty et al., 2020). Third, a neutrality plot (*P*_12_ vs. *P*_3_, where *P*_12_ is the mean of *P*_1_ and *P*_2_) was used to quantify the relative contributions of mutation and selection. The regression slope approaching 1.0 suggests a dominant role for mutation pressure, while a slope near 0 indicates stronger selection (Sueoka, 1988). RSCU patterns and all subsequent analytical plots were visualized in R v4.3.1 using the ggplot2, ggstar, and aplot packages (Wickham, 2016; Xu, 2022; Yu, 2023).

### 2.7 Phylogenetic analysis

#### 2.7.1 Maximum likelihood phylogenetic tree

In order to investigate the phylogenetic position of *Pleurozia* in relation to thalloid and leafy liverworts, we selected 35 liverwort species from the available chloroplast genome data in GenBank, representing the major orders and families of both morphological groups. Using the maximum likelihood (ML) method, a phylogenetic tree was constructed based on the chloroplast genomes of these species, with *Lunularia cruciata* serving as the outgroup. The corresponding GenBank accession numbers are listed in **Supplementary Table S1**. To assess the impact of non-coding and intronic regions on the phylogenetic tree topology, we created a dataset comprising the entire chloroplast genomes, excluding the second inverted repeat region (IRa), as noted by Xiang et al. (2022), to eliminate redundancy and enhance computational efficiency by preventing duplicate inclusion repeated consideration of identical information. For the “LSC+IRb+SSC” dataset, multiple alignments were performed using MAFFT v7.490 with the “–AUTO” strategy (Katoh et al., 2002; Katoh and Standley, 2013). Subsequently, the best-fit model for the dataset was identified using Modelfinder under the Bayesian Information Criterion (BIC), with GTR+F+R5 selected as the optimal model (Kalyaanamoorthy et al., 2017). Finally, the dataset was subjected to ML analysis using IQ-TREE multicore version 2.2.2.7 (Minh et al., 2020), employing stochastic nearest neighbor interchange (NNI) operations for tree searches and conducting 5000 ultra-fast bootstrap (BS) replications (Minh et al., 2013; Nguyen et al., 2015).

#### 2.7.2 Bayesian inference phylogenetic tree

Bayesian inference (BI) analysis was conducted using MrBayes-mpi version 3.2.7 (Huelsenbeck et al., 2001; Ronquist et al., 2012), based on the “LSC + IRb + SSC” sequences of 35 liverwort (Marchantiophyta) chloroplast genomes, with *Lunularia cruciata* as the outgroup (**Supplementary Table S1**). Sequence alignments were performed in Geneious Prime 2023.2.1 using MAFFT v7.490 with the “–auto” strategy in normal comparison mode (Katoh et al., 2002; Katoh and Standley, 2013). The optimal DNA substitution model was evaluated using MrModeltest v.2 (Nylander, 2004) under the Akaike Information Criterion (AIC), as implemented in PAUP* v.4.0a169 (Wilgenbusch and Swofford, 2003; Posada, 2003), which identified GTR + I + G as the best-fit model. The aligned sequence dataset was then conducted using Markov chain Monte Carlo (MCMC) with the following parameters: two independent runs, each with four chains (3 hot chains, 1 cold chain), running for 4 million iterations and sampling every 500 iterations. The convergence of the MCMC chains was confirmed by ensuring that the average standard deviation of split frequencies (ASDF) was less than 0.01, and the effective sample sizes (ESS) for all parameters in Tracer v1.7.2 exceeded 200 (Rambaut et al., 2018). After discarding a 10% burn-in, as determined by Tracer v.1.7.2, a majority-rule consensus tree with posterior probabilities (PP) was generated from the remaining trees.

## 3 Results

### 3.1 Chloroplast genome characteristics of *Pleurozia* species

**Figure 2:**
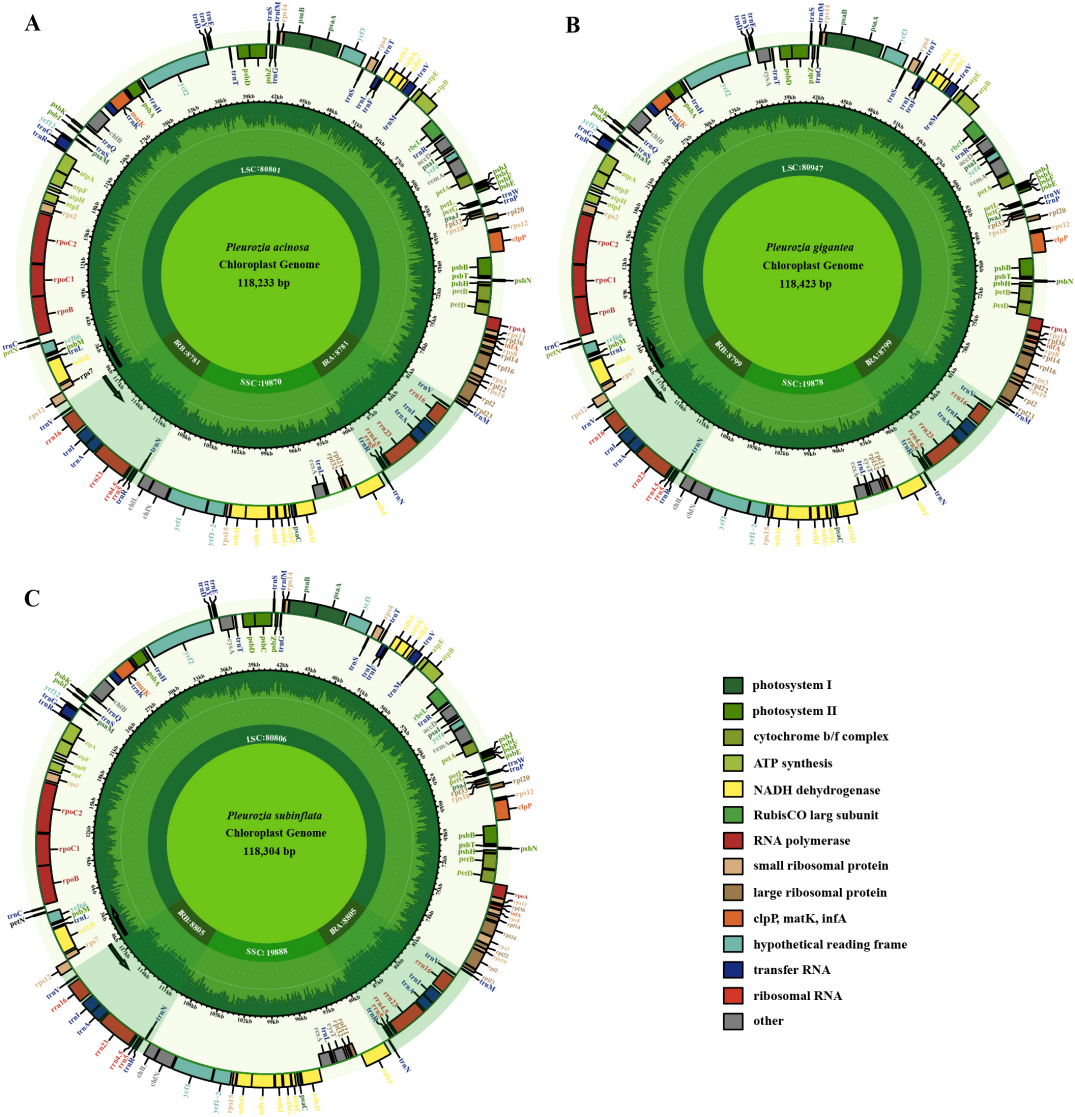
Chloroplast genome maps of *Pleurozia*. *P. acinosa* **(A)**, *P. gigantea* **(B)**, and *P. subinflata* **(C)**. Genes inside the large circles are transcribed in a clockwise direction, whereas those outside follow a counterclockwise transcriptional direction. Small circles indicate the GC content and shaded regions denote the IR areas. Genes of differing functions are distinguished by various colors.

The chloroplast genomes of the three sequenced *Pleurozia* species exhibit the same conserved quadripartite structure, comprising a large single-copy (LSC) region, a small single-copy (SSC) region, and a pair of inverted repeat (IR) regions. The genome lengths are 118,233 bp, 118,423 bp, and 118,304 bp for *P. acinosa*, *P. subinflata*, and *P. gigantea*, respectively (refer to **Table 1**; **Figure 2**). These chloroplast genomes are highly similar in gene content, overall size, and GC composition, with an average overall GC content of approximately 32%. In all three genomes, the IR regions consistently show the highest GC content (46.5–46.6%), followed by the LSC (29.9%) and SSC regions (28.6–28.8%) (**Table 1**). The annotated chloroplast genes can be categorized into three functional groups: PCGs, RNA-coding genes, and other genes, as detailed in **Table 1** and **Table 2**. Among these, 16 genes (e.g., *petB*, *petD*, *atpF*) contain single introns (**Table 2**), while two genes (*clpP* and *ycf3*) possess double introns. Notably, the *rps12* gene exhibits trans-splicing in the chloroplast genome. The IR regions contain duplicated tRNA genes, including *trnA-UGC*, *trnI-GAU*, *trnN-GUU*, *trnR-ACG*, and *trnV-GAC*. The chloroplast genome of *P. acinosa* comprises 130 genes (8 rRNA, 36 tRNA, 86 PCGs), while the other two species each possess 132 genes (8 rRNA, 36 tRNA, 88 PCGs). This difference is primarily due to the absence of the *cysA* and *cysT* genes in *P. acinosa* (refer to **Table 2**; **Figure 2**).

**Table 1:** Characteristics of cp genomes of the *Pleurozia* species.

Content	*P. acinosa*	*P. gigantea*	*P. subinflata*	*P. purpurea*
Total cp genome size (bp)	118,233	118,423	118,304	118,166
Length of inverted repeat region (bp)	8,781	8,799	8,805	8,804
Length of large single copy region (bp)	80,801	80,947	80,806	80673
Length of small single copy region (bp)	19,870	19,878	19,888	19,878
Total GC content (%)	32.1	32.2	32.2	32.2
GC content of LSC (%)	29.9	29.9	29.9	29.9
GC content of IR (%)	46.6	46.5	46.5	46.5
GC content of SSC (%)	28.6	28.7	28.7	28.8
Total number of genes	130	132	132	132
Number of tRNA genes	36	36	36	36
Number of rRNA genes	8	8	8	8
Number of protein-encoding genes	86	88	88	88

**Table 2:** List of genes annotated in three *Pleurozia* plastomes.

Gene function	Gene product	Gene
Protein-coding genes	Photosystem I	*psaA, psaB, psaC, psaI, psaJ, psaM*
	Photosystem II	*psbA, psbB, psbC, psbD, psbE, psbF, psbH, psbI, psbJ, psbK, psbL, psbM, psbN, psbT, psbZ*
	Cytochrome b/f complex	*petA*, *petB* ^a^, *petD* ^a^, *petG*, *petL*, *petN*
	ATP synthase	*atpA*, *atpB*, *atpE*, *atpF* ^a^, *atpH*, *atpI*
	NADH dehydrogenase	*ndhA* ^a^, *ndhB* ^a^, *ndhC*, *ndhD*, *ndhE*, *ndhF*, *ndhG*, *ndhH*, *ndhI*, *ndhJ*, *ndhK*
	Rubisco large subunit	*rbcL*
	Chlorophyll biosynthesis	*chlB*, *chlL*, *chlN*
	RNA polymerase	*rpoA*, *rpoB*, *rpoC1* ^a^, *rpoC2*
	Small subunit ribosomal proteins	*rps2*, *rps3*, *rps4*, *rps7*, *rps8*, *rps11*, *rps12* ^a,c^, *rps14*, *rps15*, *rps18*, *rps19*
	Large subunit ribosomal proteins	*rpl2* ^a^, *rpl14*, *rpl16* ^a^, *rpl20*, *rpl21*, *rpl22*, *rpl23*, *rpl32*, *rpl33*, *rpl36*
	Catalytic subunit of the protease	*clpP* ^b^
	Maturase	*matK*
	Translation factor	*infA*
	Acetyl-CoA carboxylase	*accD*
	Subunit A of the system II complex for C-type cytochrome biogenesis	*ccsA*
	Chloroplast envelope membrane protein	*cemA*
	Sulfate/thiosulfate import ATP-binding protein	cysA^*^, cysT^*^
Other genes	Component of TIC complex	*ycf1*, *ycf1-2*
	Component of 2-MD heteromeric AAA-ATPase complex	*ycf2*
	Hypothetical chloroplast reading frames	*ycf3* ^b^, *ycf4*, *ycf12*, *ycf66* ^a^
RNA-coding genes	Transfer RNAs	*trnA-UGC* ^a,d^, *trnC-GCA*, *trnD-GUC*, *trnE-UUC*, *trnF-GAA*, *trnfM-CAU*, *trnG-GCC*, *trnG-UCC* ^a^, *trnH-GUG*, *trnI-GAU* ^a,d^, *trnK-UUU* ^a^, *trnL-CAA*, *trnL-UAA* ^a^, *trnL-UAG*, *trnM-CAU*, *trnN-GUU* ^d^, *trnP-UGG*, *trnQ-UUG*, *trnR-ACG* ^d^, *trnR-CCG*, *trnR-UCU*, *trnS-GCU*, *trnS-GGA*, *trnS-UGA*, *trnT-GGU*, *trnT-UGU*, *trnV-GAC* ^d^, *trnV-UAC* ^a^, *trnW-CCA*, *trnY-GUA*
	Ribosomal RNAs	*rrn4.5* ^d^, *rrn5* ^d^, *rrn16* ^d^, *rrn23* ^d^

^a^Gene containing a single intron; ^b^Gene containing two introns; ^c^Trans-splicing genes; ^d^Two gene copies due to the IR; ^*^Gene deletion in species *P. acinosa*.

### 3.2 Genome comparison and nucleotide diversity

#### 3.2.1 Comparative genomic analysis

**Figure 3:**
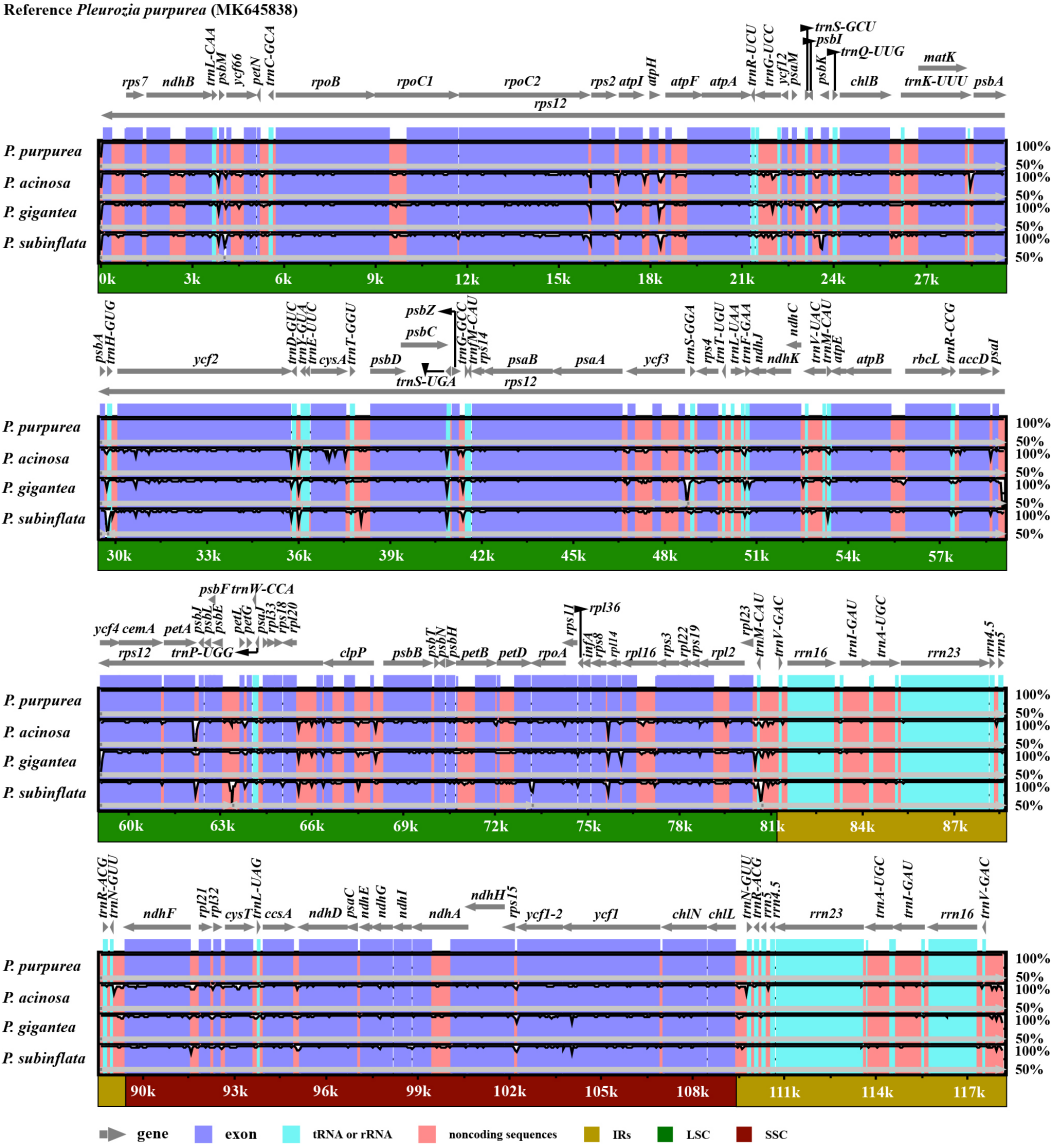
Comparative visualization of chloroplast genome sequences across four *Pleurozia* species. The y-axis represents sequence identity ranging from 50% to 100%, and the x-axis shows the position within the chloroplast genome. Arrows indicate the annotated genes and their transcription direction in the reference genome. The protein-coding and non-coding regions are highlighted in purple and orange, respectively.

mVISTA-based comparative analyses demonstrated high sequence conservation and overall similarity across *Pleurozia* chloroplast genomes (**Figure 3**). Coding regions were more conserved than non-coding regions. Non-translated elements (tRNAs, rRNAs) showed comparable levels of variation among the four species. The sequence divergence was predominantly localized in non-coding and intronic regions. Notable intron variations were identified in *ycf66*, *trnG-UCC*, *ycf3*, *trnL-UAA*, *trnV-UAC*, and *clpP*. Highly variable non-coding regions included *psbM*~*ycf66*, *atpH*~*atpF*, *psbI*~*psbK*, *psbA*~*trnH-GUG*, *trnD-GUC*~*trnY-GUA*, *psbC*~*trnS-UGA*, *ycf3*~*trnS-GGA*, *psbZ*~*trnG-GCC*, *petA*~*psbJ*, *psbE*~*petL*, *rpl20*~*rps12*, *clpP*~*psbB*, *trnM-CAU*~*trnV-GAC*, and *chlL*~*trnN-GUU*.

#### 3.2.2 Genome collinearity analysis and nucleotide diversity

**Figure 4:**
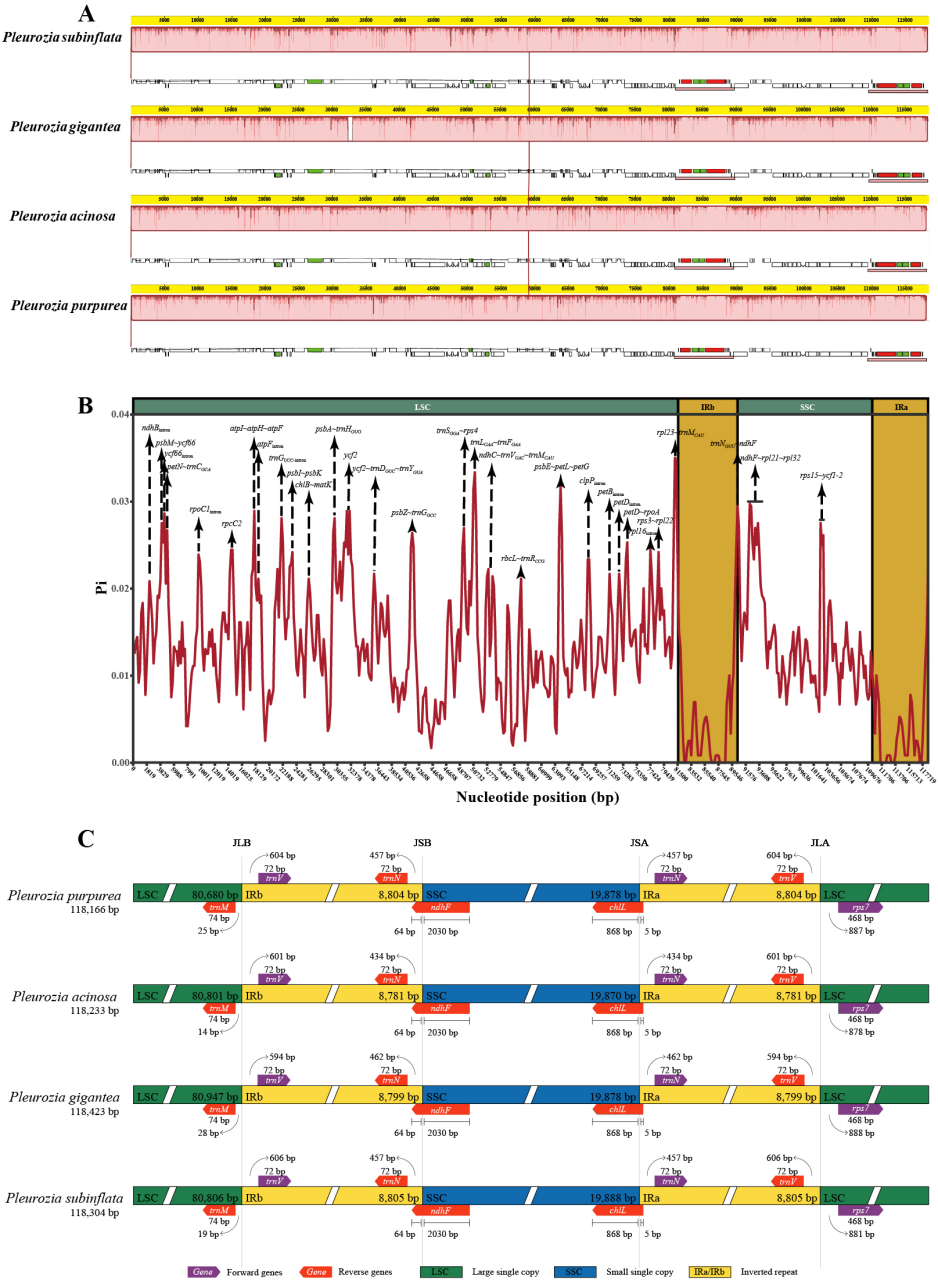
Comparative genomics analyses of four *Pleurozia* species. **(A)** Mauve alignment of four *Pleurozia* plastomes. Locally co-linear blocks are represented by continuous colored regions. **(B)** Sliding window analysis of the whole chloroplast genomes across *Pleurozia* species. Step length: 600 bp; window length: 200 bp. **(C)** Comparative analysis of the junctions between the IR regions and two single copy regions (LSC/SSC) in four *Pleurozia* chloroplast genomes. Colored boxes above or below the main line indicate adjacent border genes. The distance between the genes and boundaries are represented by the base lengths (bp). JLA, junction between LSC and inverted repeat (IRA). JLB, junction between LSC and IRB. JSA, junction between SSC and IRA. JSB, junction between SSC and IRB.

Genome collinearity analyses demonstrated structural conservation among the chloroplast genomes of *Pleurozia* species (**Figure 4A**). The absence of gene inversions or genomic rearrangements confirms high collinearity among these genomes. Nucleotide diversity (Pi) analyses revealed Pi (π) values ranging from 0 to 0.0350, with a genome-wide average of 0.0127 (**Figure 4B**). The IR regions exhibited significantly lower variability compared to the LSC and SSC regions. Three hypervariable non-coding regions (π > 0.03) were identified: *rpl23*~*trnM-CAU* (0.0350), *psbE*~*petL*~*petG* (0.0317), and *trnL-UAA*~*trnF-GAA* (0.0314). Additionally, 15 moderately variable non-coding regions (e.g., *trnN-GUU*~*ndhF*, *atpI*~*atpH*~*atpF*, *psbA*~*trnH-GUG*) and six intronic regions (*ycf66*, *trnG-UCC*, *rpl16*, *rpoC1*, *clpP*, *ndhB* introns) were detected (see **Figure 4B**; **Supplementary Table S2**). Notably, *ycf2* was the only moderately variable protein-coding region.

#### 3.2.3 IR contraction and expansion analysis

As depicted in **Figure 4C**, the chloroplast genomes of the four *Pleurozia* species exhibit highly conserved SC/IR boundary architecture, with no significant expansions or contractions. Notably, the *ndhF* gene extends across the IRb/SSC junction, with 64 bp located within IRb region, while the *chlL* gene spans the IRa/SSC boundary, containing merely 5 bp into the IRa region.

### 3.3 Selective pressure analysis

**Figure 5:**
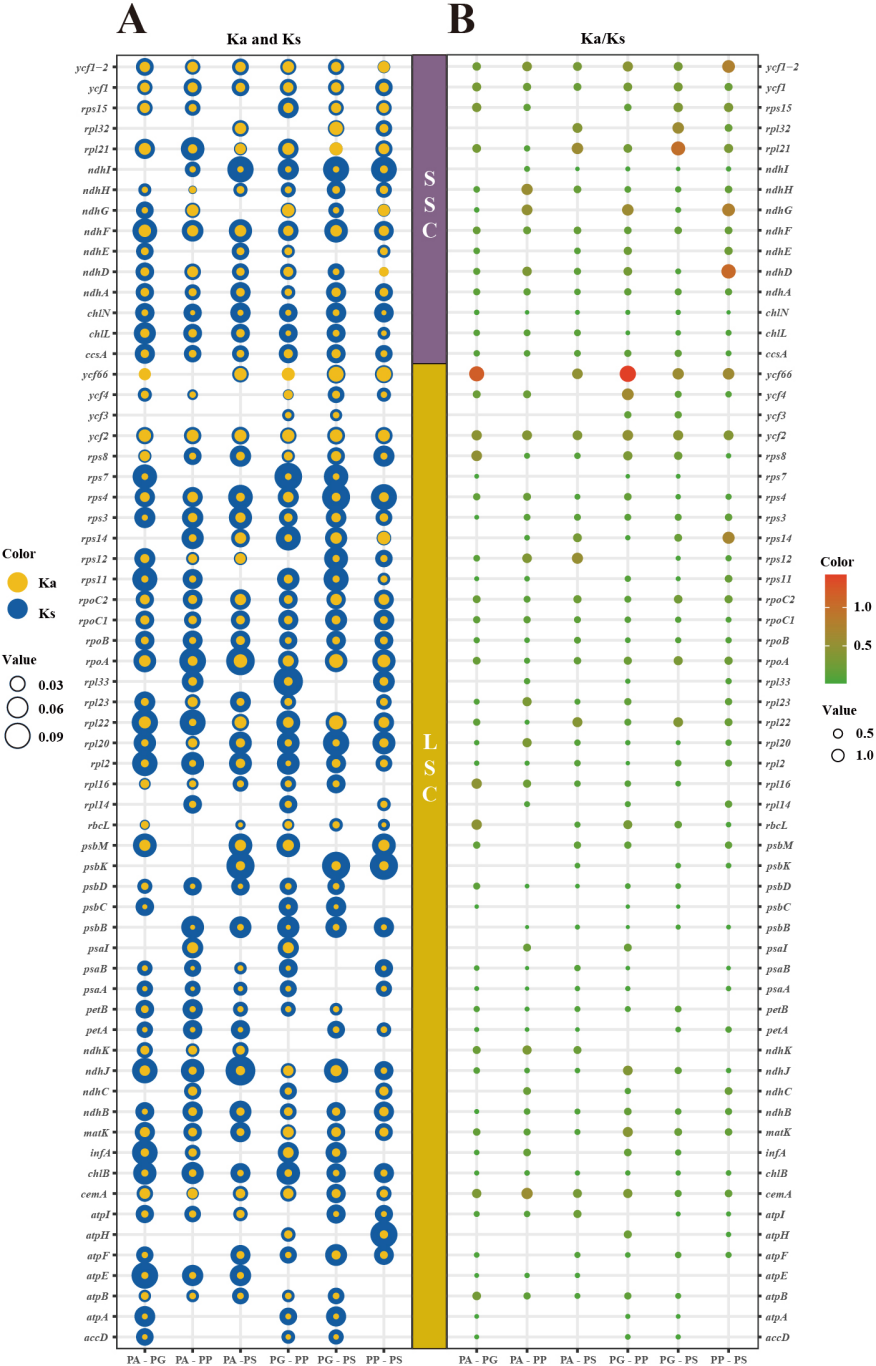
Bubble plots are employed to illustrate the variation in selection pressure across 63 PCGs in four *Pleurozia* chloroplast genomes. **(A)** Ka (blue bubbles) and Ks (yellow bubbles) substitution rates. Bubble size corresponds to Ka or Ks values; yellow predominance indicates Ka/Ks > 1. **(B)** Ka/Ks ratios. Bubble size and color intensity reflect ratio magnitude; larger ratios indicate stronger selection pressure. Vertical axis: individual genes; horizontal axis: species pairs (PA, *P. acinosa*; PG, *P. gigantea*; PP, *P. purpurea*; PS, *P. subinflata*).

In the chloroplast genomes across four *Pleurozia* species, only *ycf66* and *ndhD* exhibited Ka/Ks > 1, indicating positive selection. The remaining genes displayed ratios < 1 (**Figure 5**), consistent with genome-wide purifying selection. These findings underscore strong evolutionary constraints in *Pleurozia* chloroplast genomes, reflecting the conservation of essential protein functions through the selective removal of deleterious mutations.

### 3.4 Codon usage bias of chloroplast genomes of *Pleurozia* species

#### 3.4.1 GC content of each codon position

A total of 58, 60, 60, and 60 CDS from *P. acinosa*, *P. gigantea*, *P. purpurea*, and *P. subinflata* were analyzed, respectively. The results showed no significant interspecific variation in overall GC content (**Supplementary Table S3**). However, significant heterogeneity (*p* < 0.0001) was observed among GC1, GC2, and GC3, demonstrating a consistent pattern of GC1(43.40%) > GC2 (36.75%) > GC3 (18.04%) across all species (**Supplementary Figure S1**; **Supplementary Table S3**).

#### 3.4.2 RSCU analysis

**Figure 6:**
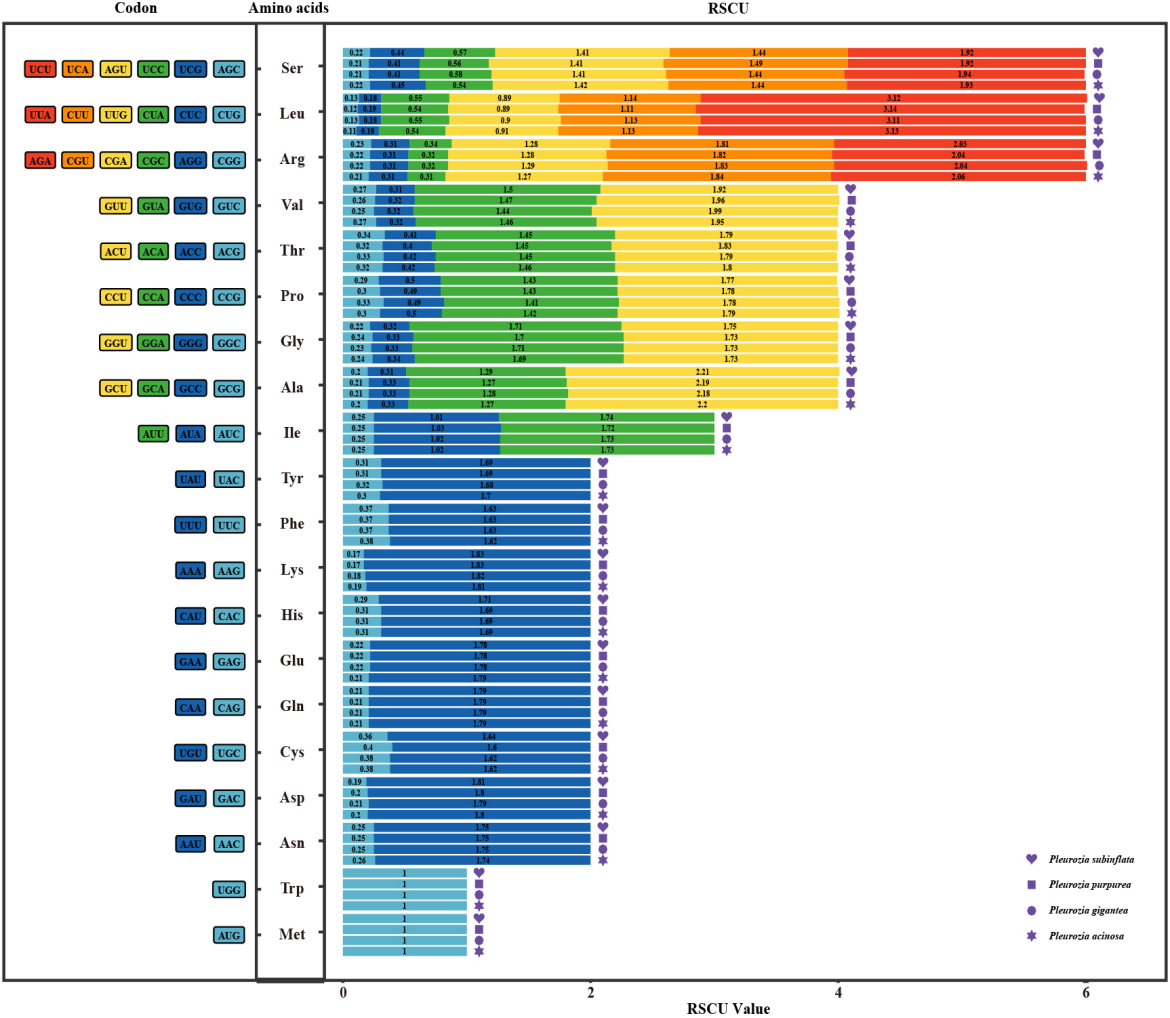
RSCU-plot in the chloroplast genomes of four *Pleurozia* species. Codons are listed on the left side of the graph, corresponding amino acids in the center, and RSCU values on the right.

The analyses identified 29 codons with RSCU values >1 (**Supplementary Table S4**), including 16 U-ending and 13 A-ending codons, demonstrating a pronounced preference for U/A-terminated codons in the chloroplast genomes of *Pleurozia.* Notably, the Leucine-encoding UUA codon exhibited an RSCU value exceeding 3. As shown in **Figure 6**, the RSCU ratios are broadly similar across the four *Pleurozia* species, indicating a consistent codon usage pattern.

#### 3.4.3 ENC plot analysis

**Figure 7:**
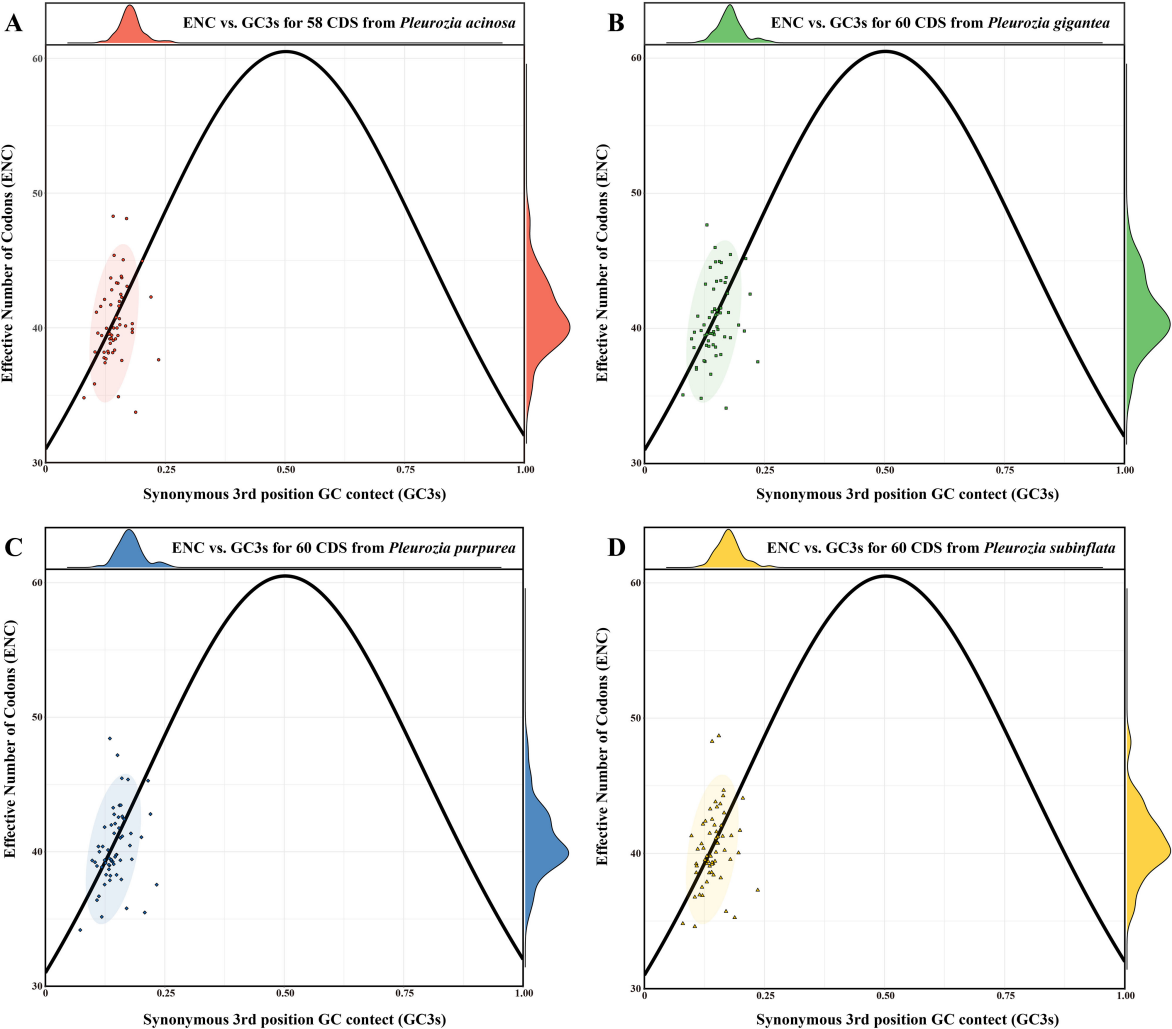
ENC-GC3s plot of the chloroplast genomes of *Pleurozia* species. **(A–D)** ENC-GC3s results of *P. acinosa*, *P. gigantea*, *P. purpurea*, and *P. subinflata*, respectively. The circles represent 95% confidence intervals.

The ENC values ranged from 33.74 to 48.28 in *P. acinosa* (mean = 40.42), 37.10 to 47.66 in *P. gigantea* (mean = 40.56), 34.18 to 48.42 in *P. purpurea* (mean = 40.30), and 34.59 to 48.69 in *P. subinflata* (mean = 40.34). All genes exhibited ENC values below 50, with species means exceeding 35 (**Figures 7A–D**), indicating moderate CUB in *Pleurozia* chloroplast genomes. The ENC plots for the four *Pleurozia* species (**Figures 7A–D**) revealed dispersed distributions relative to the expected curve. While a minority of genes clustered near the standard curve, the majority of genes were scattered around it. This pattern suggests synergistic effects of mutation pressure and natural selection in shaping CUB. Furthermore, no significant interspecific differences were observed in ENC values, GC3s composition (**Supplementary Figures S2A–B**), or overall codon usage patterns (**Supplementary Figure S2C–D**).

#### 3.4.4 PR2-plot analysis

**Figure 8:**
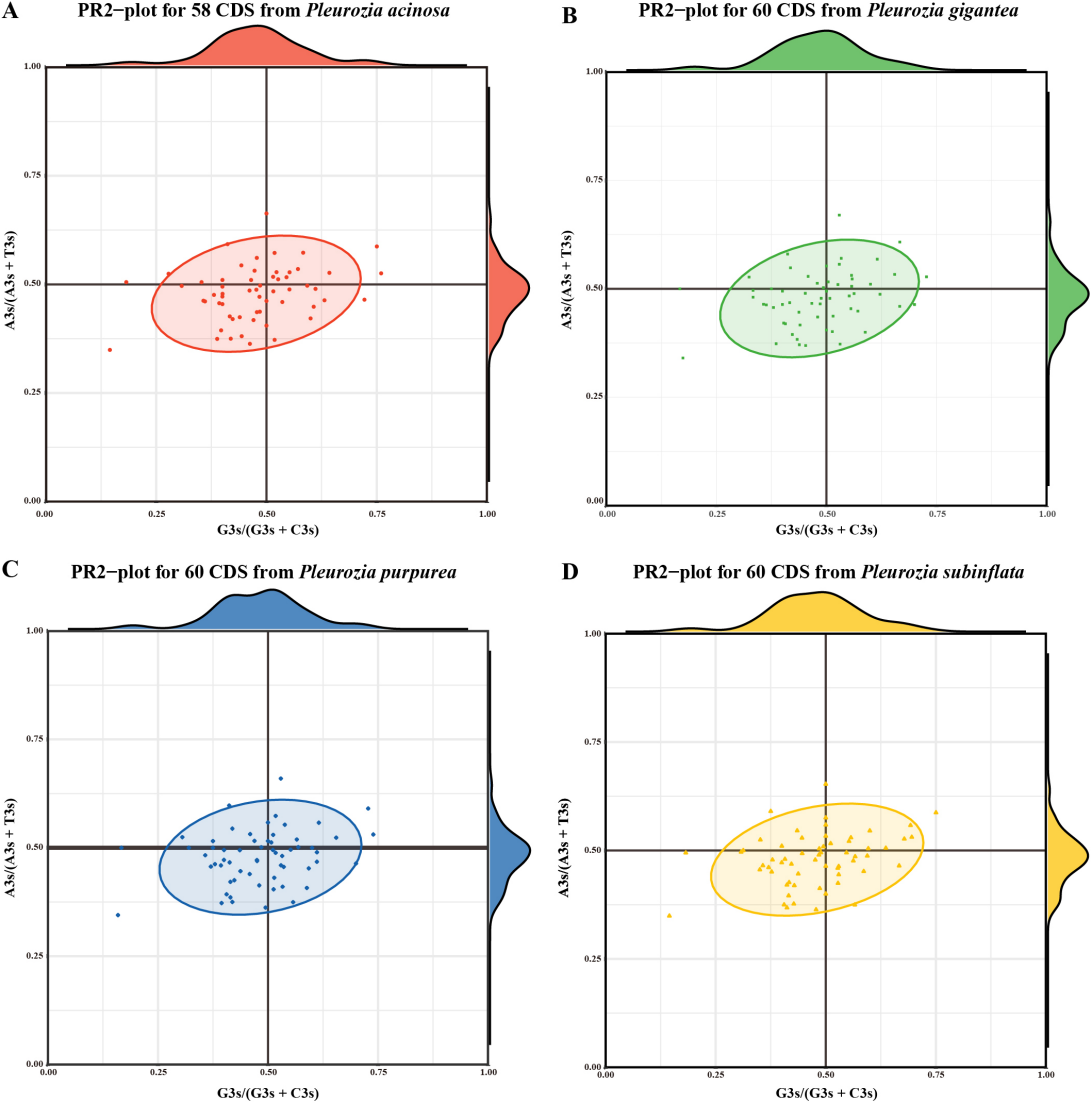
PR2-plot analysis of chloroplast genomes in *Pleurozia* species. **(A–D)** PR2-plot results for *P. acinosa*, *P. gigantea*, *P. purpurea*, and *P. subinflata*, respectively. Density profiles of G3s/(G3s + C3s) and A3s/(A3s + T3s) in A-D are shown on the top and right edges, respectively.

The PR2-plot results (**Figures 8A–D**) revealed asymmetric CDS distribution across all four *Pleurozia* species, with CDS points predominantly clustered in the lower-left quadrant (A3s < T3s, G3s < C3s), indicating a clear preference for T/C bases. A small number of CDS approached the plot center (A3s/(A3s + T3s) ≈ 0.5, G3s/(G3s + C3s) ≈ 0.5), suggesting limited neutral evolution dynamics. The consistent, unbalanced usage of bases at the third codon position across *Pleurozia* species highlights conserved evolutionary constraints mediated by the combined effects of mutational pressures, selective constraints, and other forces.

#### 3.4.5 Neutrality-plot analysis

**Figure 9:**
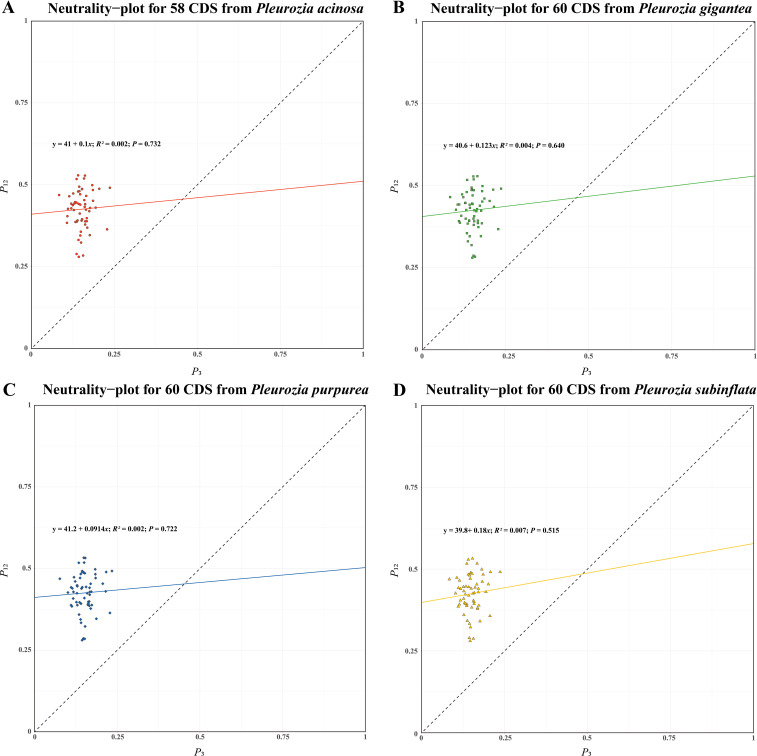
Neutral-plot analysis of CUB in the chloroplast genomes of *Pleurozia* species. **(A–D)** Results for *P. acinosa*, *P. gigantea*, *P. purpurea*, and *P. subinflata*, respectively. Horizontal axis: *P*_3_ value; vertical axis: *P*_12_ value.

As shown in **Figures 9A–D**, the regression analysis revealed that only a few genes were diagonally distributed in the plot, and *P*_12_ exhibited no significant correlation with *P*_3_ (r for all species < 0.09, P > 0.05), suggesting that natural selection may exert a considerable influence on the CUB of the four *Pleurozia* species. Furthermore, the slopes of the regression lines were 0.1000 (*P. acinosa*), 0.1230 (*P. gigantea*), 0.0914 (*P. purpurea*) and 0.1800 (*P. subinflata*), indicating that the mutation pressure across the four species accounted for only 9.14% to 18.00%. Consequently, these results imply that natural selection is superior to mutation pressure in shaping the development of CUB in *Pleurozia*.

### 3.5 Phylogenetic analysis of *Pleurozia* chloroplast genomes

**Figure 10:**
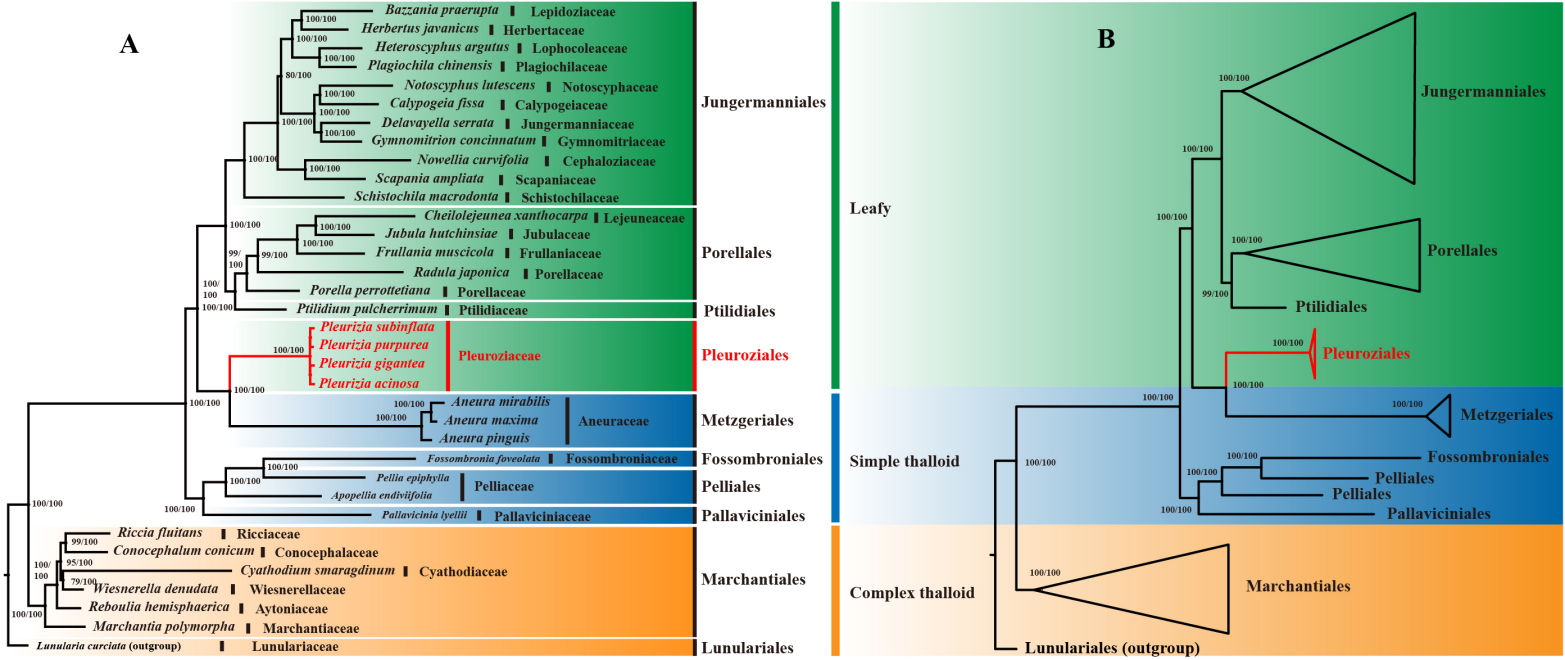
Topological comparison and Consensus phylogenetic trees for 35 Liverwort species constructed using ML and BI methods. **(A)** Topological comparison trees. **(B)** Order-level phylogenetic tree. The BS and PP values are annotated at each node to indicate statistical confidence. The red font highlights the order *Pleuroziales*.

Phylogenetic analyses of 35 liverwort chloroplast genomes (LSC + IRb + SSC regions) using both maximum likelihood (ML) and Bayesian inference (BI) methods revealed highly congruent topologies. Most nodes corresponding to orders and families received strong support, with ML bootstrap (BS) values ≥ 80 and Bayesian posterior probabilities (PP) of 1.00 (**Figures 10A, B**). On the resulting phylogenetic tree, the Marchantiales clade, together with Lunulariales, belongs to the complex thalloid liverworts. The remaining taxa form a major clade, further divided into two subclades. One consists of Pallaviciniales, Pelliales, and Fossombroniales, representing a group of simple thalloid liverworts. The other contains Metzgeriales, another group of simple thalloid liverworts, along with the leafy liverwort orders Pleuroziales, Ptilidiales, Porellales, and Jungermanniales. The four *Pleurozia* species form a strongly supported monophyletic group representing Pleuroziales (BS/PP = 100/1.00), which is resolved as the sister lineage to Metzgeriales.

## 4 Discussion

### 4.1 Chloroplast genome features and genome variations

The genus *Pleurozia* represents a pivotal lineage in the evolutionary transition from thalloid to leafy liverworts, offering significant insights into the liverwort phylogeny (He-Nygrén et al., 2006). In this study, we present three complete chloroplast genomes from *Pleurozia* species, along with the first comparative genomic analysis of this genus. The findings reveal a conserved quadripartite chloroplast genome architecture, consisting of one SSC region, one LSC region, and two IR regions (**Figure 2**), a typical organization found in most green plants, including bryophytes (Dong et al., 2021; Xiang et al., 2022). The chloroplast genome lengths of the four *Pleurozia* species range from 118,116 to 118,432 bp, aligning with the genome sizes observed in other Marchantiophyta species (Ślipiko et al., 2020; Dong et al., 2021; Sawicki et al., 2021), yet shorter than those of earlier-diverging hornworts (Yu et al., 2019; Xiang et al., 2022). Comparative analyses demonstrate a high degree of structural conservation and complete collinearity (**Figure 4A**), with minimal fluctuations at the IR boundaries (**Figure 4C**). Nucleotide diversity patterns indicate mutation rate hotspots primarily in the LSC and SSC regions, in contrast to the highly conserved IR regions. This pattern is consistent with observations across land plants (Hu et al., 2023; Xiao et al., 2023). The hypervariable regions are mainly located in non-coding and intronic sequences (**Figure 4B**), paralleling evolutionary patterns observed in angiosperms (Xiao and Ge, 2022; Yang et al., 2022). These highly variable loci exhibit more polymorphic sites than standard DNA barcodes, demonstrating their potential as phylogenetic markers for resolving complex taxonomic relationships (Ren et al., 2021; Hu et al., 2023).

### 4.2 Genome annotation and deletion of *cysA* and *cysT* genes

Our annotation of the three *Pleurozia* species identified 130–132 chloroplast genes per genome, including eight rRNA, 36 tRNA, and 86–88 PCGs (**Table 2**; **Figure 2**). Notably, *P. acinosa* uniquely lacks the *cysA* and *cysT* genes, which are typically present in this genus. This co-deletion also occurs in other members of Jungermanniopsida, such as *Fossombronia cristula*, *Pallavinicia lyellii*, and *Metzgeria leptoneura* (Wicke et al., 2011; Dong et al., 2021). The *cysA* and *cysT* genes exhibit a patchy distribution across bryophytes, being variably present in hornworts (Kugita, 2003), liverworts (Ohyama et al., 1986; Wicke et al., 2011; Dong et al., 2021), and mosses (Sadamitsu et al., 2021). This irregular pattern suggests that these genes have been independently lost multiple times during bryophyte evolution (Wicke et al., 2011). Typically located in the chloroplasts, *cysA* and *cysT* are integral components of the sulfate transport system, facilitating the uptake of sulfate—a crucial nutrient for plant growth and development—from the external environment into the cell and its distribution within the plant (Cackett et al., 2022). However, studies have shown that species lacking *cysA* and *cysT* can still transport cysteine via nuclear-encoded proteins (Kopriva et al., 2008). The evolutionary pattern of *cysA* and *cysT* loss remains unclear, and such deletions have not previously been reported in *Pleurozia*. This study presents the first documented case of *cysA* and *cysT* co-deletion in *Pleurozia*, contributing to a broader understanding of recurrent gene loss in bryophyte evolution (Dong et al., 2021).

### 4.3 Selection pressure

Ka/Ks ratios are widely used to assess evolutionary pressures on PCGs, enabling the differentiation between purifying selection, neutral drift, and positive selection (Yang and Bielawski, 2000; Raman et al., 2022). Non-synonymous substitutions (Ka) result in amino acid changes that can affect protein structure and function, potentially contributing to adaptive evolution. In contrast, synonymous substitutions (Ks) do not alter the amino acid sequence and are generally considered selectively neutral, thereby serving as a baseline for estimating mutation rates (Hurst, 2002). A Ka/Ks ratio < 1 suggests purifying selection, reflecting the preferential elimination of deleterious non-synonymous mutations. Conversely, a Ka/Ks > 1 indicates positive selection, signifying the preferential retention of advantageous mutations that promote adaptive genetic changes and accelerate gene evolution (Navarro and Barton, 2003). In *Pleurozia*, the *ycf66* and *ndhD* genes exhibit Ka/Ks ratios greater than 1, suggesting that they are subject to positive selection, which may drive adaptive changes in these loci. In contrast, most chloroplast genes display Ka/Ks ratios below 1, indicating they are under purifying selection that acts to preserve their functional integrity. This pattern aligns with the general evolutionary conservation of chloroplast genomes in angiosperms, as exemplified by *Epimedium* (Wang et al., 2023).

### 4.4 Codon usage bias

Plants generally exhibit a high GC content. In nuclear coding regions, monocots tend to prefer codons ending in C or G, whereas dicots display a higher frequency of codons ending in U or A (Parvathy et al., 2022). In contrast, chloroplast and mitochondrial genomes typically show a preference for codons ending in U or A. This codon usage bias has been observed in studies of chloroplast genomes across various plant species, such as *Oryza* (Chakraborty et al., 2020) and *Elaeagnus* (Li et al., 2023). In bryophytes, including *Pleurozia*, the chloroplast genomes generally exhibit lower GC content compared to those of most angiosperms (Yu et al., 2019). There is a clear preference for U/A-terminated codons in the chloroplast genome of *Pleurozia*. Comparative analyses of CUB across the *Pleurozia* chloroplast genomes (**Figures 6**–**9**) revealed consistent patterns among the four species. ENC-GC3s plots displayed gene distributions both near and far from the expected curve, reflecting that codon usage in *Pleurozia* is shaped by a combination of mutational pressures and natural selection. The similar pattern was previously reported in the mitochondrial genome of *P. purpurea* (Wang et al., 2010). PR2 plots revealed a preference for T and C at the third codon positions, likely reflecting adaptive fine-tuning of translational efficiency superimposed on inherent mutational tendencies (Quax et al., 2015). This dual evolutionary mechanism allows for limited codon adaptation while maintaining genomic stability (Parvathy et al., 2022). Furthermore, neutral plot analyses suggest that natural selection exerts a stronger influence than mutation in determining codon usage patterns in *Pleurozia*. This trend mirrors observations in most angiosperms, where CUB is influenced by both natural selection and mutational pressure, with natural selection playing the dominant role (Yang et al., 2023).

### 4.5 Phylogenetic analysis

As the earliest diverging lineages of land plants, liverworts, mosses, and hornworts occupy a pivotal position in plant phylogeny, providing key insights into the evolutionary transition from aquatic to terrestrial environments (Wang et al., 2022). In this study, the phylogenetic tree (**Figure 10**) comprises major orders representing complex thalloid, simple thalloid, and leafy liverworts. Pleuroziales, a monotypic order containing only the genus *Pleurozia*, forms a distinct monophyletic lineage. It is resolved as the sister group to Metzgeriales, a representative of simple thalloid liverworts. Together, these two lineages cluster with the leafy liverworts. Although Pleuroziales exhibits a leafy morphology, its close phylogenetic affinity with Metzgeriales suggests a stronger evolutionary relationship with simple thalloid liverworts. This relationship is consistent with their shared characteristic of possessing a two-sided apical cell (Thiers, 1993).

The phylogeny also indicates that simple and complex thalloid liverworts belong to separate clades, with simple thalloid group showing closer affinity to leafy liverworts. Notably, the simple thalloid liverworts are divided into two groups: one forms a monophyletic clade, while Metzgeriales alone clusters sister to the leafy liverworts. Among the leafy liverworts, all orders except Pleuroziales comprise a distinct clade. This topology aligns with previous studies (Dong et al., 2021; Xiang et al., 2022; Li et al., 2024) and strongly supports *Pleurozia*’s transitional position between simple thalloid and leafy liverworts, confirming earlier hypotheses (Forrest et al., 2006; Dong et al., 2021; Shen et al., 2025). These findings contribute to both the taxonomic and genomic knowledge of *Pleurozia* and enhance our understanding of liverwort evolution.

## 5 Conclusions

In our study, we sequenced the complete chloroplast genomes of three *Pleurozia* species and provided comprehensive insights into their structural and evolutionary dynamics. The genomes exhibit a conserved quadripartite structure with minimal interspecific variation in overall size, gene content, and boundary architecture. While coding regions were highly conserved, non-coding sequences and introns displayed notable variability, particularly within three hypervariable regions, highlighting their potential as informative markers for species delimitation and phylogenetic analysis. The lower variation in IR regions compared to single-copy regions supports their proposed stabilizing role in chloroplast genome evolution. Codon usage bias (CUB) analyses revealed that natural selection plays a predominant role over mutation pressure in shaping codon preferences across all *Pleurozia* species, with a pronounced preference for U/A-ended codons. The combined effects of natural selection and mutation pressure contribute to maintaining moderate CUB, balancing translational efficiency with genomic stability. Notably, purifying selection was predominant across protein-coding regions, while only ycf66 and ndhD exhibited signs of positive selection, underscoring the strong functional constraints governing chloroplast genome evolution. Phylogenetic reconstruction robustly supports *Pleurozia* as a monophyletic clade, placing it in an intermediate evolutionary position between thalloid and leafy liverworts. This finding aligns with morphological evidence and previous phylogenetic hypotheses, highlighting the transitional role of *Pleurozia* in liverwort diversification.

## Data Availability

The datasets presented in this study can be found in online repositories. The names of the repository/repositories and accession number(s) can be found below: https://www.ncbi.nlm.nih.gov/genbank/, OR168937 https://www.ncbi.nlm.nih.gov/genbank/, OR168938 https://www.ncbi.nlm.nih.gov/genbank/, OR168939.
