# Supplementary material for: Complete chloroplast genomes of three Pleurozia species and comparative analyses with P. purpurea: codon usage bias and phylogeny
Source: Front Plant Sci. 2025 Jul 18;16:1599291. doi: 10.3389/fpls.2025.1599291 (PMC12313706; doi:10.3389/fpls.2025.1599291)
Supplement: Supplementary file 1 [file Presentation1.pdf]

Supplementary Material

1 Supplementary Figures and Tables

1.1 Supplementary Figures

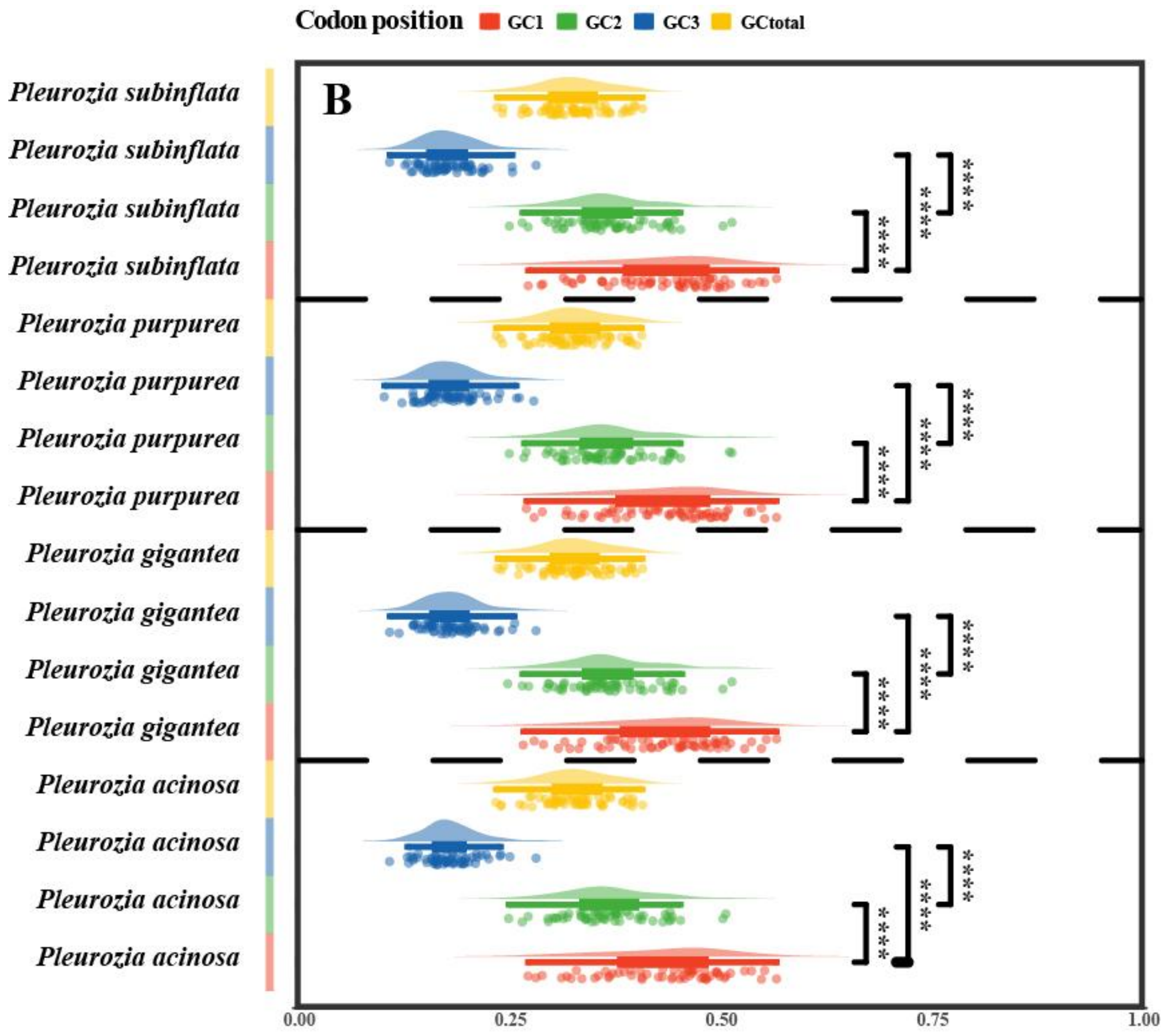

Supplementary Figure 1. Comparison of GC content in the chloroplast genomes of four *Pleurozia* species.

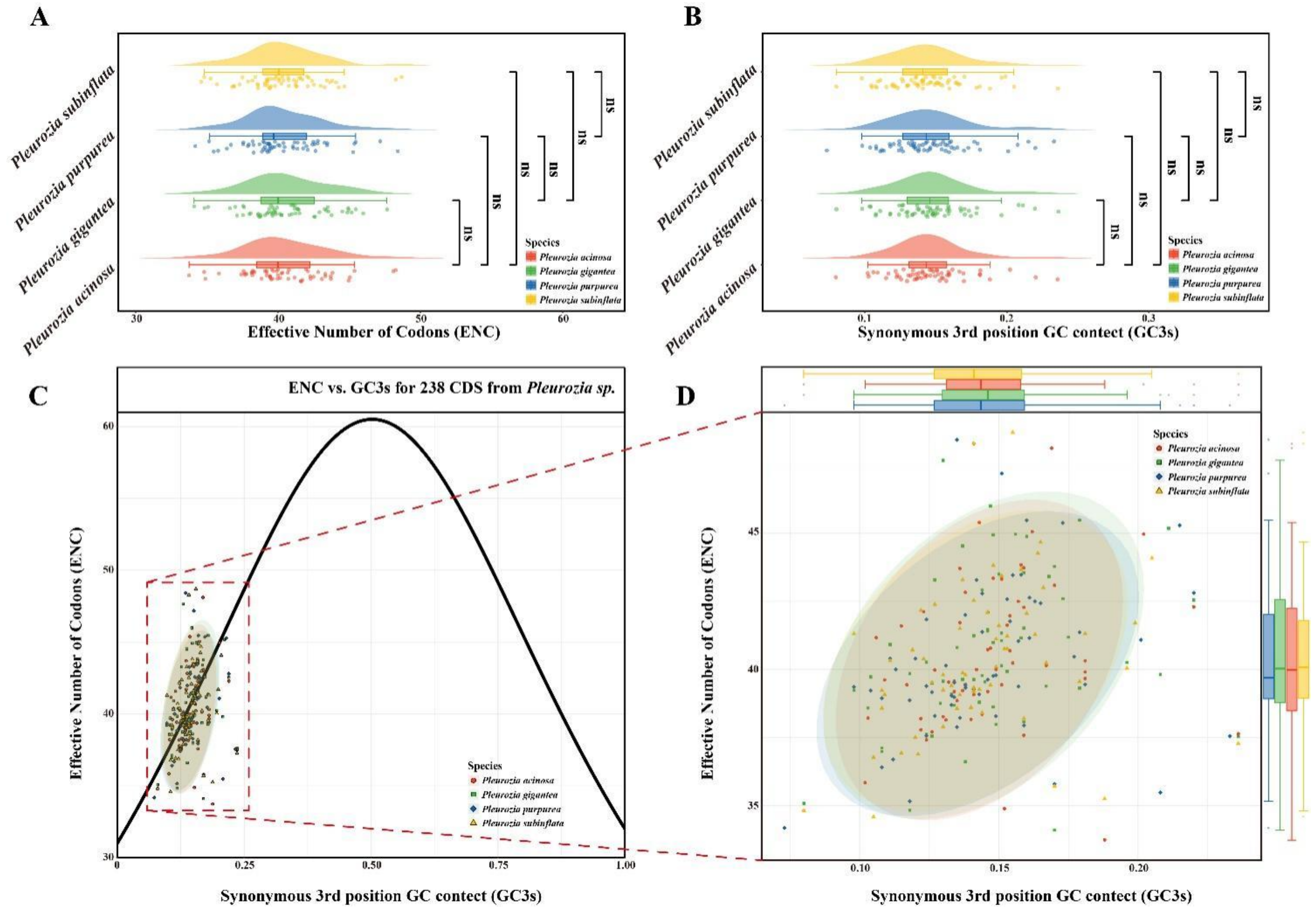

Supplementary Figure 2. ENC-GC3s plot of the chloroplast genomes of *Pleurozia* species. A, B: Differences in ENC, GC3s among the four *Pleurozia* species, respectively. C, D: Combined ENC-GC3s differences for all four *Pleurozia* species. The circles represent 95% confidence intervals.

1.2 Supplementary Tables

Supplementary Table 1. Information of 35 liverwort species used to construct phylogenetic trees

No.	GenBank number	Species	Genus	Family	Order
1	MW429511	*Lunularia curciata*	*Lunularia*	Lunulariaceae	Lunulariales
2	NC_042505	*Marchantia polymorpha*	*Marchantia*	Marchantiaceae	Marchantiales
3	NC_042418	*Reboulia hemisphaerica*	*Reboulia*	Aytoniaceae	Marchantiales
4	NC_050394	*Wiesnerella denudata*	*Wiesnerella*	Wiesnerellaceae	Marchantiales
5	MT023024	*Conocephalum conicum*	*Conocephalum*	Conocephalaceae	Marchantiales
6	NC_042887	*Riccia fluitans*	*Riccia*	Ricciaceae	Marchantiales
7	MW429509	*Cyathodium smaragdinum*	*Cyathodium*	Cyathodiaceae	Marchantiales
8	NC_058632	*Pallavicinia lyellii*	*Pallavicinia*	Pallaviciniaceae	Pallaviciniales
9	OR220796	*Apopellia endiviifolia*	*Apopellia*	Pelliaceae	Pelliales
10	NC_080367	*Pellia epiphylla*	*Pellia*	Pelliaceae	Pelliales
11	NC_080365	*Fossombronia foveolata*	*Fossombronia*	Fossombroniaceae	Fossombroniales
12	NC_082503	*Aneura maxima*	*Aneura*	Aneuraceae	Metzgeriales
13	NC_010359	*Aneura mirabilis*	*Aneura*	Aneuraceae	Metzgeriales
14	NC_035617	*Aneura pinguis*	*Aneura*	Aneuraceae	Metzgeriales
15	OR168937	*Pleurozia acinosa*	*Pleurozia*	Pleuroziaceae	Pleuroziales
16	OR168938	*Pleurozia gigantea*	*Pleurozia*	Pleuroziaceae	Pleuroziales
17	OR168939	*Pleurozia subinflata*	*Pleurozia*	Pleuroziaceae	Pleuroziales
18	MK645838	*Pleurozia purpurea*	*Pleurozia*	Pleuroziaceae	Pleuroziales
19	NC_015402	*Ptilidium pulcherrimum*	*Ptilidium*	Ptilidiaceae	Ptilidiales
20	NC_043780	*Porella perrottetiana*	*Porella*	Porellaceae	Porellales
21	NC_043781	*Radula japonica*	*Radula*	Radulaceae	Porellales
22	NC_079849	*Frullania muscicola*	*Frullania*	Frullaniaceae	Porellales
23	NC_043782	*Jubula hutchinsiae*	*Jubula*	Jubulaceae	Porellales
24	NC_043777	*Cheilolejeunea xanthocarpa*	*Cheilolejeunea*	Lejeuneaceae	Porellales
25	NC_043779	*Schistochila macrodonta*	*Schistochila*	Schistochilaceae	Jungermanniales
26	NC_051002	*Scapania ampliata*	*Scapania*	Scapaniaceae	Jungermanniales
27	NC_043784	*Plagiochila chinensis*	*Plagiochila*	Plagiochilaceae	Jungermanniales
28	MW429513	*Notoscyphus lutescens*	*Notoscyphus*	Notoscyphaceae	Jungermanniales
29	NC_043788	*Heteroscyphus argutus*	*Heteroscyphus*	Lophocoleaceae	Jungermanniales
30	NC_043785	*Bazzania praerupta*	*Bazzania*	Lepidoziaceae	Jungermanniales
31	MW429508	*Delavayella serrata*	*Delavayella*	Jungermanniaceae	Jungermanniales
32	MW429507	*Herbertus javanicus*	*Herbertus*	Herbertaceae	Jungermanniales
33	NC_040133	*Gymnomitrion concinnatum*	*Gymnomitrion*	Gymnomitriaceae	Jungermanniales
34	NC_063926	*Nowellia curvifolia*	*Nowellia*	Cephaloziaceae	Jungermanniales
35	NC_043787	*Calypogeia fissa*	*Calypogeia*	Calypogeiaceae	Jungermanniales

Supplementary Table 2. Variable regions with large Pi values in sliding window analysis

Variable regions	Pi value	Genomic region
rps23 ~ trnM-CAU	0.03500	LSC
psbE ~ petL ~ petG	0.03167	LSC
trnL-UAA ~ trnF-GAA	0.03139	LSC
trnN-GUU ~ ndhF	0.02944	SSC
atpI ~ atpH ~ atpF	0.02889	LSC
ycf66 intron	0.02861	LSC
psbA ~ trnH-GUG	0.02806	LSC
trnG-UCC intron	0.02806	LSC
psbM ~ ycf66	0.02750	LSC
ndhF ~ rpl21 ~ rpl32	0.02694	SSC
trnS-GGA ~ rps4	0.02694	LSC
petN ~ trnC-GCA	0.02667	LSC
psbZ ~ trnG-GCC	0.02639	LSC
petD ~ rpoA	0.02528	LSC
ycf2	0.02528	LSC
rps15 ~ ycf1-2	0.02500	SSC
rpl16 intron	0.02444	LSC
rps3 ~ rpl22	0.02417	LSC
psbI ~ psbK	0.02417	LSC
rpoC1 intron	0.02389	LSC
clpP intron	0.02333	LSC
ndhC ~ trnV-UAC ~ trnM-CAU	0.02222	LSC
rbcL ~ trnR-CCG	0.02111	LSC
chlB ~ matK	0.02111	LSC
ndhB intron	0.02083	LSC

Supplementary Table 3. GC content in chloroplast genomes of four *Pleurozia* species.

Species	GC1	GC2	GC3	GCall
Pleurozia acinosa	0.4343 ± 0.0719	0.3681 ± 0.0538	0.1806 ± 0.0321	0.3277 ± 0.0412
Pleurozia gigantea	0.4340 ± 0.0715	0.3668 ± 0.0538	0.1818 ± 0.0331	0.3276 ± 0.0411
Pleurozia purpurea	0.4314 ± 0.0707	0.3674 ± 0.0539	0.1806 ± 0.0340	0.3273 ± 0.0410
Pleurozia subinflata	0.4336 ± 0.0709	0.3675 ± 0.0533	0.1786 ± 0.0328	0.3266 ± 0.0411
Pleurozia spp.	0.4340 ± 0.0708	0.3675 ± 0.0534	0.1804 ± 0.0328	0.3273 ± 0.0409

Supplementary Table 4. Overall RSCU of the cp genomes of four *Pleurozia* species

Amino acid	Genetic codon	P. acinosa	P. gigantea	P. purpurea	P. subinflata	Amino acid	Genetic codon	P. acinosa	P. gigantea	P. purpurea	P. subinflata
Ala	**GCA**	1.27	1.28	1.27	1.29	Leu	CUA	0.54	0.55	0.54	0.55
	GCC	0.33	0.33	0.33	0.31		CUC	0.18	0.18	0.19	0.18
	GCG	0.20	0.21	0.21	0.2		CUG	0.11	0.13	0.12	0.13
	**GCU**	2.20	2.18	2.19	2.21		**CUU**	1.13	1.13	1.11	1.14
Arg	**AGA**	2.06	2.04	2.04	2.03		**UUA**	3.13	3.11	3.14	3.12
	AGG	0.31	0.31	0.31	0.31		UUG	0.91	0.90	0.89	0.89
	**CGA**	1.27	1.29	1.28	1.28	Lys	**AAA**	1.81	1.82	1.83	1.83
	CGC	0.31	0.32	0.32	0.34		AAG	0.19	0.18	0.17	0.17
	CGG	0.21	0.22	0.22	0.23	Phe	UUC	0.38	0.37	0.37	0.37
	**CGU**	1.84	1.83	1.82	1.81		**UUU**	1.62	1.63	1.63	1.63
Asn	AAC	0.26	0.25	0.25	0.25	Pro	**CCA**	1.42	1.41	1.43	1.43
	**AAU**	1.74	1.75	1.75	1.75		CCC	0.50	0.49	0.49	0.5
Asp	GAC	0.20	0.21	0.20	0.19		CCG	0.30	0.33	0.30	0.29
	**GAU**	1.80	1.79	1.80	1.81		**CCU**	1.79	1.78	1.78	1.77
Cys	UGC	0.38	0.38	0.40	0.36	Ser	AGC	0.22	0.21	0.21	0.22
	**UGU**	1.62	1.62	1.60	1.64		**AGU**	1.42	1.41	1.41	1.41
Gln	**CAA**	1.79	1.79	1.79	1.79		**UCA**	1.44	1.44	1.49	1.44
	CAG	0.21	0.21	0.21	0.21		UCC	0.54	0.58	0.56	0.57
Glu	**GAA**	1.79	1.78	1.78	1.78		UCG	0.45	0.41	0.41	0.44
	GAG	0.21	0.22	0.22	0.22		**UCU**	1.93	1.94	1.92	1.92
Gly	**GGA**	1.69	1.71	1.70	1.71	Thr	**ACA**	1.46	1.45	1.45	1.45
	GGC	0.24	0.23	0.24	0.22		ACC	0.42	0.42	0.40	0.41
	GGG	0.34	0.33	0.33	0.32		ACG	0.32	0.33	0.32	0.34
	**GGU**	1.73	1.73	1.73	1.75		**ACU**	1.80	1.79	1.83	1.79
His	CAC	0.31	0.31	0.31	0.29	Tyr	UAC	0.30	0.32	0.31	0.31
	**CAU**	1.69	1.69	1.69	1.71		**UAU**	1.70	1.68	1.69	1.69
Ile	**AUA**	1.02	1.02	1.03	1.01	Val	**GUA**	1.46	1.44	1.47	1.50
	AUC	0.25	0.25	0.25	0.25		GUC	0.27	0.25	0.26	0.27
	**AUU**	1.73	1.73	1.72	1.74		GUG	0.32	0.32	0.32	0.31
Met	AUG	1.00	1.00	1.00	1.00		**GUU**	1.95	1.99	1.96	1.92
Trp	UGG	1.00	1.00	1.00	1.00						

Preferred codons for *Pleurozia* are in bold italics.
